# Histopathologic assessment of cultured human thymus

**DOI:** 10.1371/journal.pone.0230668

**Published:** 2020-03-24

**Authors:** Laura P. Hale, Jadee Neff, Lynn Cheatham, Diana Cardona, M. Louise Markert, Joanne Kurtzberg

**Affiliations:** 1 Department of Pathology, Duke University School of Medicine, Durham, NC, United States of America; 2 Marcus Center for Cellular Cures, Duke University School of Medicine, Durham, NC, United States of America; 3 Department of Pediatrics, Duke University School of Medicine, Durham, NC, United States of America; Wake Forest Institute for Regenerative Medicine, UNITED STATES

## Abstract

The maintenance and propagation of complex mixtures of cells *in vitro* in the form of native organs or engineered organoids has contributed to understanding mechanisms of cell and organ development and function which can be translated into therapeutic benefits. For example, allogeneic cultured postnatal human thymus tissue has been shown to support production of naïve recipient T cells when transplanted into patients with complete DiGeorge anomaly and other genetic defects that result in congenital lack of a thymus. Patients receiving such transplants typically exhibit reversal of their immunodeficiency and normalization of their peripheral blood T cell receptor V-beta repertoire, with long-term survival. This study was designed to assess the histopathologic changes that occur in postnatal human thymus slices when cultured according to protocols used for transplanted tissues. Results showed that as thymic organ cultures progressed from days 0 through 21, slices developed increasing amounts of necrosis, increasing condensation of thymic epithelium, and decreasing numbers of residual T cells. The architecture of the thymic epithelial network remained generally well-preserved throughout the 21 days of culture, with focal expression of cytokeratin 14, a putative biomarker of thymic epithelial cells with long-term organ-repopulating potential. All organ slices derived from the same donor thymus closely resembled one another, with minor differences in size, shape, and relative content of cortex versus medulla. Similarly, slices derived from different donors showed similar histopathologic characteristics when examined at the same culture time point. Taken together, these results demonstrate that diagnostic criteria based on structural features of the tissue identifiable via hematoxylin and eosin staining and cytokeratin immunohistochemistry can be used to evaluate the quality of slices transplanted into patients with congenital athymia.

## Introduction

Patients with complete DiGeorge anomaly and other forms of congenital athymia have severe immunodeficiency due to their lack of a thymus and the resulting absence of T-cell production. Transplantation of cultured postnatal thymus tissue has been shown to successfully support production of naïve T cells in patients with congenital athymia due to DiGeorge anomaly and other genetic causes (e.g. deficiency of TBX2 or FOXN1) [1–8]. Transplanted patients develop naïve peripheral blood T cells with normalization of the peripheral blood T cell receptor V-beta repertoire and immune function [5,9]. Biopsy of thymus tissue 2–4 months after transplantation demonstrates colonization of donor thymus stroma by recipient thymocytes, with normal-appearing cortex and medulla and Hassall bodies [10,11]. Overall survival in transplanted patients is 72% (43/60) at 1 year and 73% (36/49) at 2 years. In contrast, almost all untreated patients die by age 2 due to overwhelming infection [5,7,12,13]. The mechanism by which thymic transplantation restores immune function in these patients is hypothesized to be due to signaling stimulated by direct contact of thymic epithelial cells in the transplanted thymus with recipient T cell precursors that leads to generation of mature functional T cells.

Thymus tissue intended for transplantation is obtained with permission from the parent(s) of immunocompetent infant donors undergoing cardiac surgery. The donated tissue then undergoes a series of processing steps that include slicing and culture for 12 to 21 days [5,11]. The purpose of culture is to partially deplete T cells from the thymic epithelial network. This depletion provides space for colonization of the depleted thymus slices by recipient T cell precursors and also minimizes the potential for graft-versus-host disease mediated by donor T cells. This study describes the histopathologic changes that occur during the culture of thymus slices. Understanding these changes can potentially lead to the validation of enhanced histopathologic criteria for prospective assessment of the quality of cultured thymus slices prior to transplantation, based on characteristics of tissues that have successfully generated immune reconstitution in prior recipients [12].

## Materials and methods

Thymic tissue was obtained from immunocompetent infant donors <9 months of age who were undergoing corrective cardiac surgery where removal of a portion of the thymus was routinely required to facilitate the cardiac repair. The parent(s) of each donor provided written informed consent to allow any thymic tissue that was removed and otherwise would be discarded to be potentially used for transplantation or research. These studies were approved by the Institutional Review Board of Duke University Medical Center. The donor thymus used for the research portion of this study was sliced and cultured in a Good Manufacturing Process (GMP)-compliant cell manufacturing laboratory using donor qualification and culture procedures identical to those used for thymus samples intended for transplantation [2,3,5,14,15]. However, rather than transplantation into recipients, all slices from these research lots were fixed in 10% neutral buffered formalin at specific time points during culture and entirely submitted for histopathologic examination. All slices from 5 thymuses were submitted at a single time point at either day 0 (56 slices), day 5 (23 slices), day 9 (28 slices), day 12 (21 slices), or 21 (12 slices). For the remaining 3 thymuses, 1/5 of the slices representing that thymus were submitted for examination at each of days 0, 5–7, 9, 12, or 20–21 (n = 28 total slices per time point). Fixed slices were submitted to a Clinical Laboratory Improvement Act (CLIA)-certified, College of American Pathologists (CAP)-accredited clinical laboratory, where formalin-fixed paraffin embedded blocks, hematoxylin and eosin (H&E)-stained sections, and an immunohistochemical panel were prepared using procedures identical to those used to assess lots intended for transplantation. Antibodies used included pan-cytokeratin (clones AE1/AE3; Leica), cytokeratin (CK) 14 (clone LL02; Leica), CD3 (clone LN10; Leica), and Ki-67 (clone MIB-1; Dako). Automated immunohistochemistry was performed using standard immunoperoxidase methodologies and 3, 3’-diaminobenzidine (brown) substrate, with a hematoxylin counterstain. Some of these slices were also evaluated for reactivity with antibodies specific for terminal deoxynucleotidyl transferase (clone TdT; clones SEN28; Leica), CD45RO (clone UCHL1; Dako), CD43 (clone DF-T1; Dako), and the T cell receptor β chain (βF1, clone 8A3, Thermo Scientific). H&E and immunohistochemical slides (AE1/AE3, CK14, CD3, Ki-67) were also examined from 11 lots of cultured thymus that were implanted into athymic recipients contemporaneously and up to 2.5 years before preparation of the research lots described above.

## Results

### Initial identification of tissue as thymus

**Fig 1:**
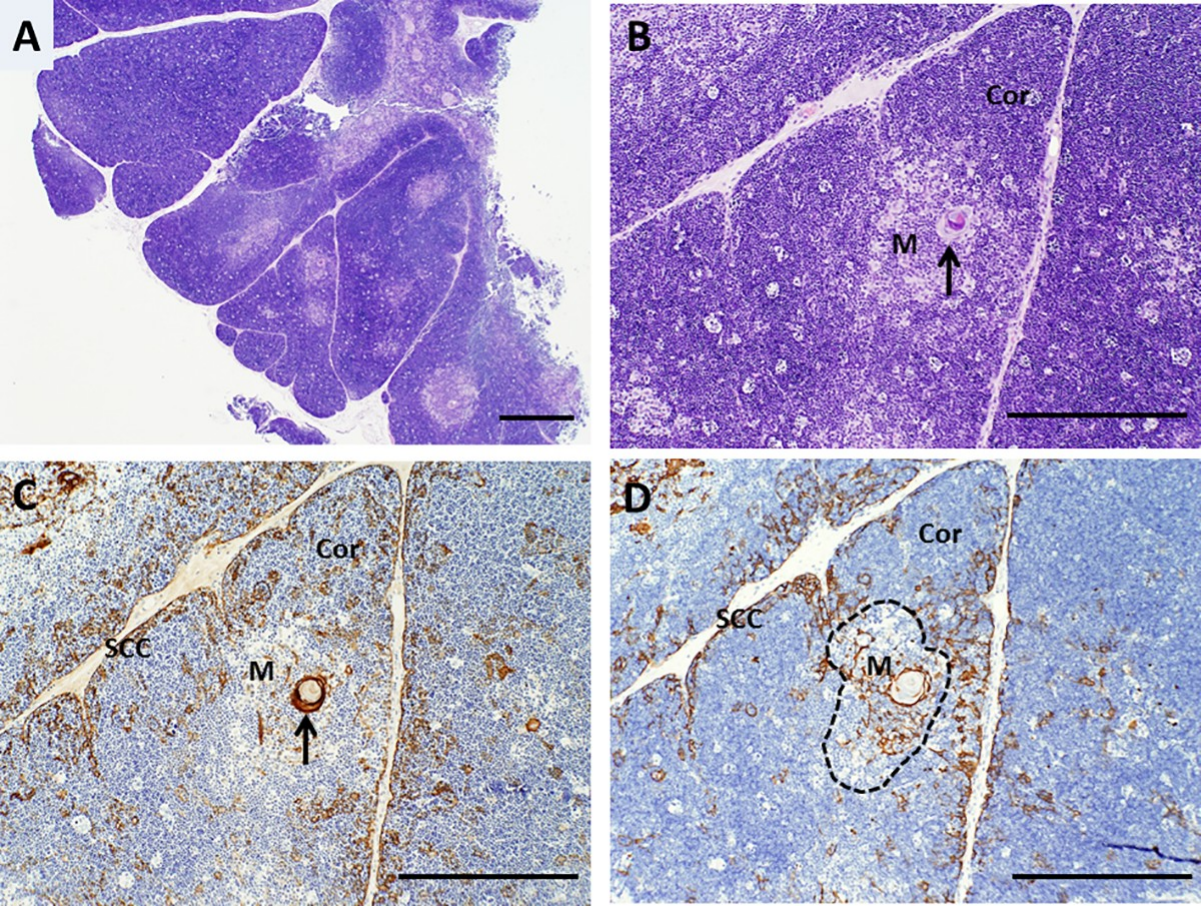
Architecture of cultured thymus, day 0. A, B. Hematoxylin and eosin (H&E) staining shows well-defined cortical and lighter-staining medullary areas, as expected for normal pediatric thymus. C. Immunohistochemistry with a cocktail of antibodies (AE1/AE3) that together detect all types of epithelial cells demonstrates that thymic epithelial cells are present beneath the capsule and in a light lacy network in both cortex and medulla. Arrows in B and C point to a Hassall body. D. Cytokeratin 14 (CK14) antibody reacts with thymic epithelial cells in the sub-capsular cortex and in the medulla, as well as with scattered thymic epithelial cells in the cortex. The dotted line highlights an area of medulla that is surrounded by cortex. Brown color indicates a positive reaction with antibody. SCC denotes sub-capsular cortex, Cor denotes cortex, and M denotes medulla. Bar represents 1 mm in A and 500 μm in B–D.

The thymus is a bi-lobed organ that is present in the anterior mediastinum. It possesses a thin connective tissue capsule that is typically removed during processing within the laboratory. However, extensions of the capsule (trabeculae) and associated blood vessels, fibrous tissue, adipose tissue, and variable numbers of mature hematopoietic cells may still be observed in thymus slices. The trabeculae divide the thymus tissue into lobules that are composed of a lacy-appearing three-dimensional network of thymic epithelial cells with intervening spaces containing the developing T cells (thymocytes). The lobules typically demonstrate an outer cortex and an inner medulla, which vary in their histologic appearance as well as phenotype and function of the cells they contain. The thymic cortex is very basophilic (blue) due to densely packed immature thymocytes that stain very darkly with hematoxylin. The density of the more mature thymocytes that are present in the thymic medulla is lower than that of the cortex, so the medulla tends to appear more eosinophilic (pink). The thymic medulla also contains pathognomonic eosinophilic cytokeratin-containing structures called Hassall bodies. Macrophages present within the thymus may form “tingible bodies” that appear as a light- colored circular area against a background of darkly staining thymocytes (a “starry sky” pattern). Large numbers of tingible bodies are characteristic of stress involution, which may occur in normal thymus donors due to the stress of severe cardiac defects, surgery, and/or corticosteroid treatment and does not disqualify a thymus for transplantation. The typical histologic features of sliced normal thymus prior to culture (Day 0) are shown in **Fig 1**.

### Assessment of Hassall bodies

The thymic medulla contains characteristic structures called Hassall bodies that are composed of terminally differentiated thymic epithelial cells that react with monoclonal antibodies that also react with the terminally differentiated upper layers of the epidermis (reviewed in [16]).

**Fig 2:**
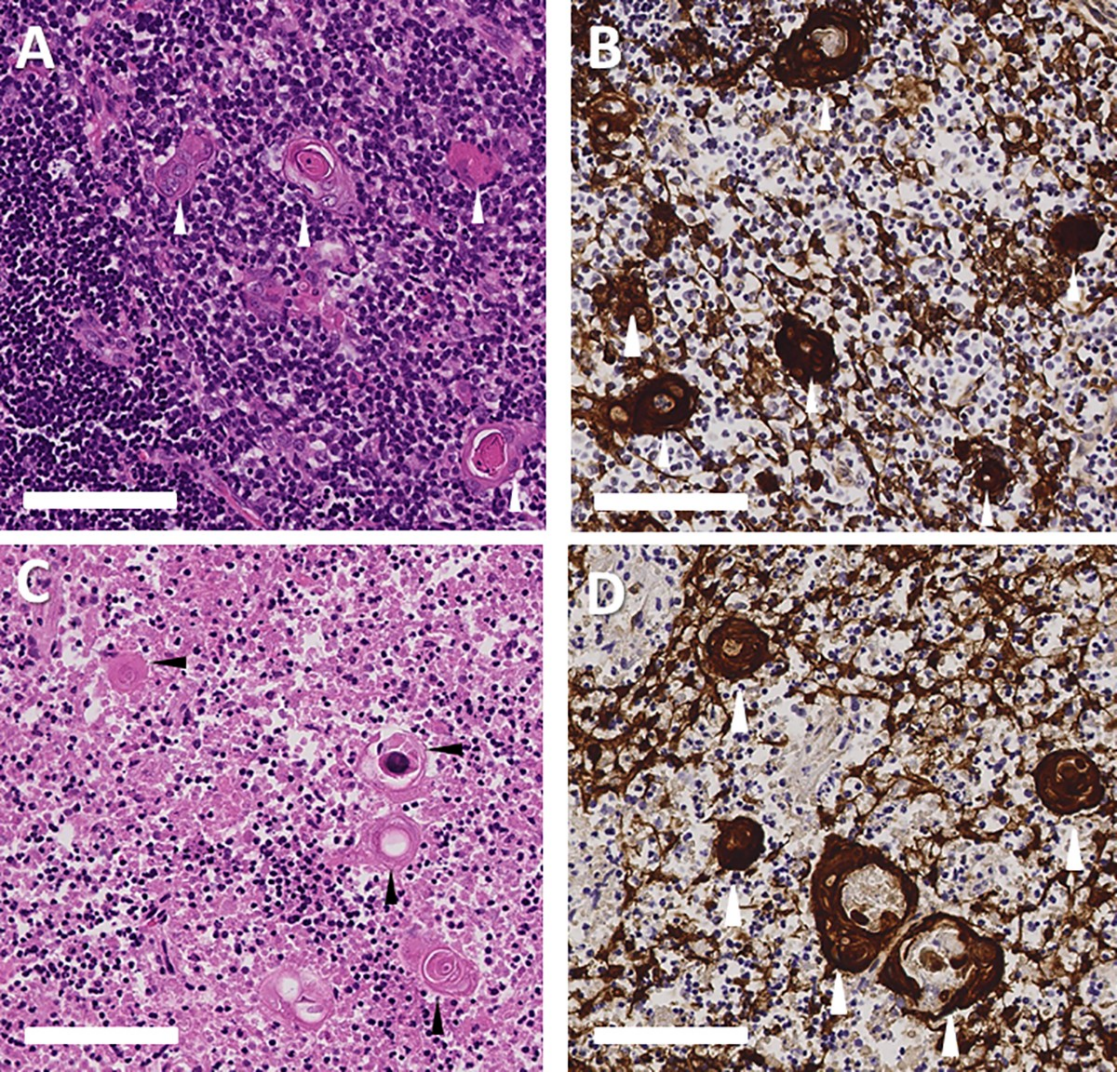
Examples of Hassall bodies. The histologic appearance of Hassall bodies is shown on day 0 (A, B) and day 9 (C, D) of culture. Hematoxylin and eosin (H&E) stain is shown in panels A and C. Reactivity with pan-cytokeratin (AE1/AE3) antibodies is shown in panels B and D; brown color indicates a positive reaction. Arrowheads point out representative Hassall bodies, which appear less prominent on H&E-stained sections of cultured thymus due to the depletion and necrosis of the surrounding thymocytes. However, Hassall bodies can still be readily identified by careful examination or by using immunohistochemistry. Bar represents 100 μm.

Hassall bodies may serve as a marker of tissue identity, since they are found only in thymus. They may also reflect the quality of the input donor tissue, since the terminal differentiation process is normally triggered by thymocyte-thymic epithelial cell interactions [16]. Hassall bodies are usually readily identified by their appearance as eosinophilic whorls of epithelial cells on H&E-stained slides (**Fig 2**). The central-most layers typically lack nuclei. Variable amounts of cellular debris may also be present in the center of the Hassall bodies. Hassall bodies stain very strongly with AE1/AE3 antibodies, which can be used as secondary confirmation of the identity of these structures if not definitively identified in H&E-stained slides.

### General thymocyte changes during culture

**Fig 3:**
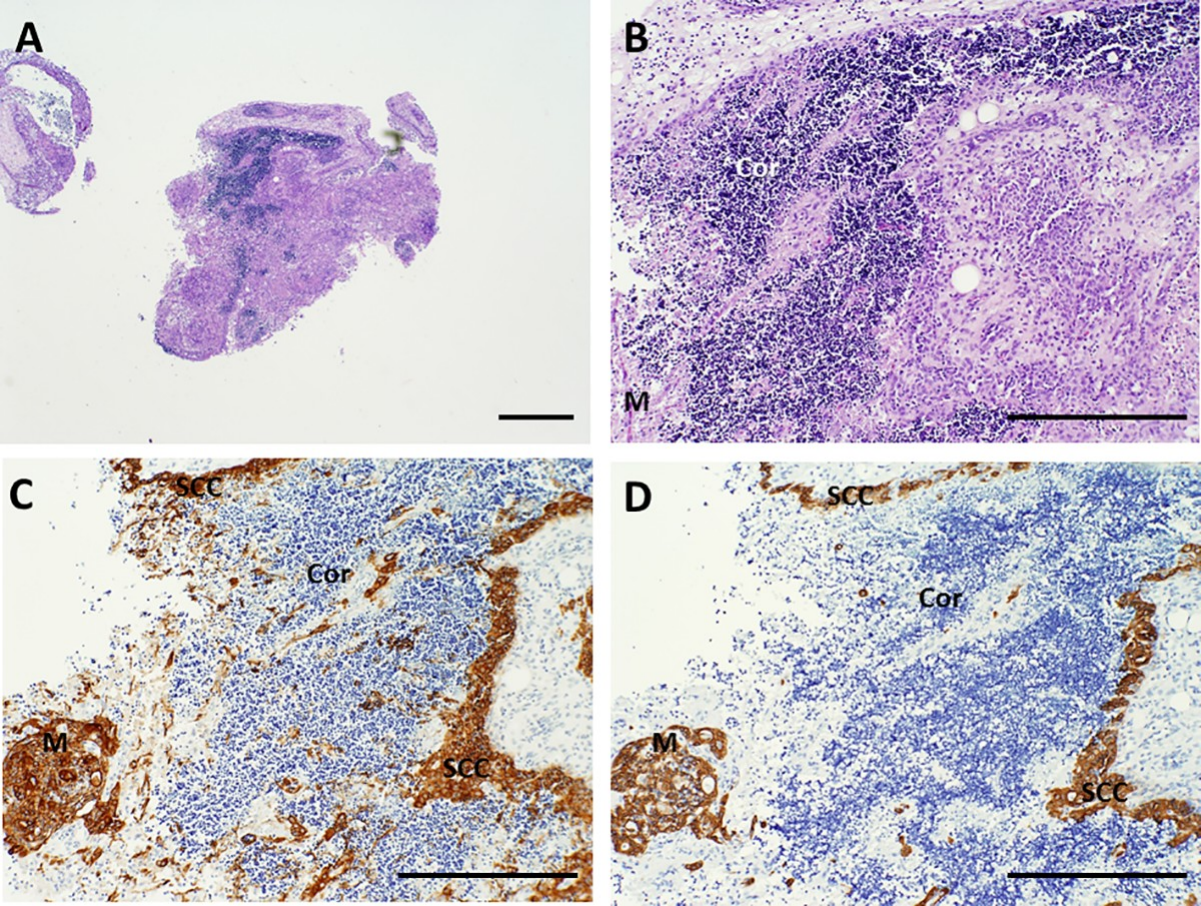
Architecture of cultured thymus, day 7. A, B. Hematoxylin and eosin (H&E) stain shows marked depletion of thymocytes, although some cortical areas (Cor) still contain large numbers of thymocytes with retained nuclei. Pan-cytokeratin (AE1/AE3) (C) and cytokeratin 14 (CK14) immunohistochemistry (D) show condensation of the thymic epithelium in the subcapsular cortex (SCC) and in the medulla (M). Brown color indicates a positive reaction with antibody. Bar represents 1 mm in A and 500 μm in B–D.

**Fig 4:**
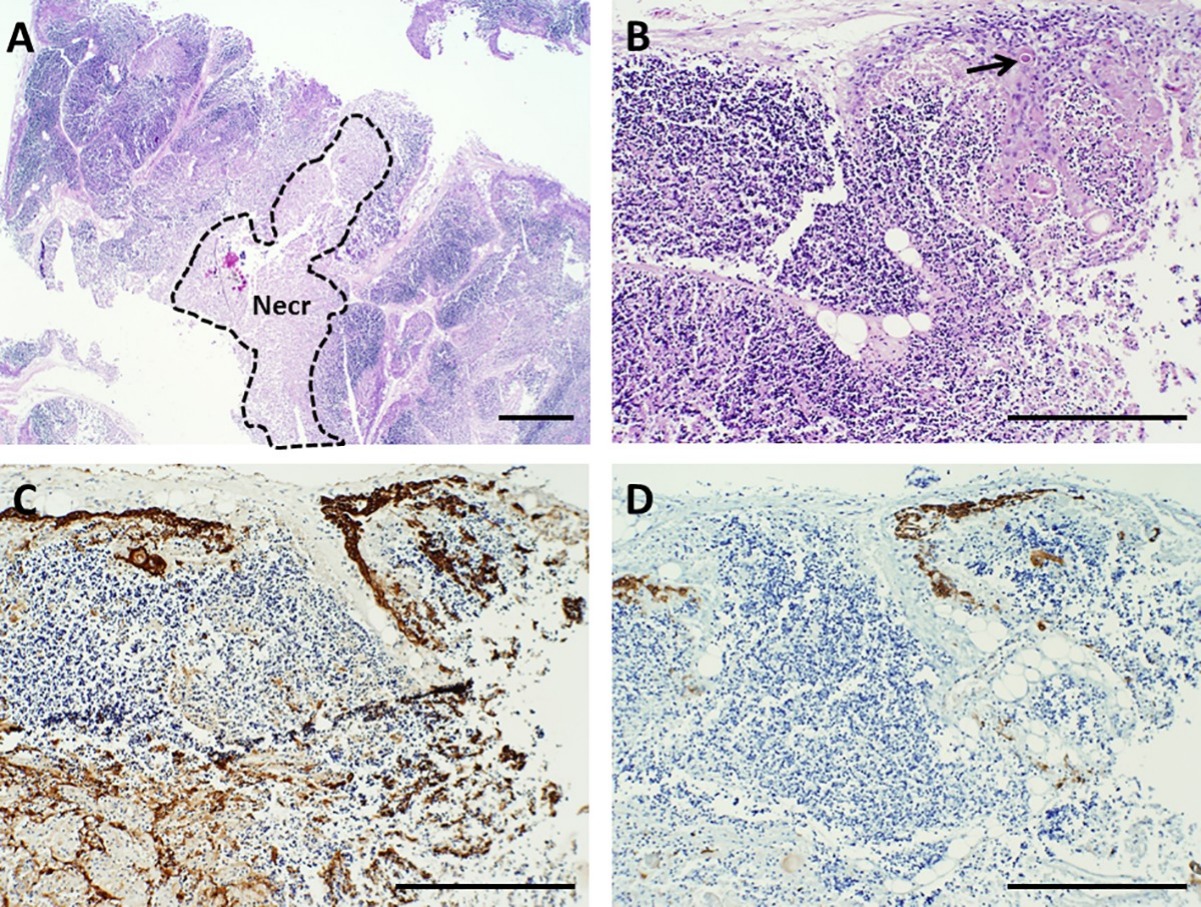
Architecture of cultured thymus, day 9. A, B. Hematoxylin and eosin (H&E) stain. Few if any live T cells or thymic epithelial cells are present in the pale-staining area in A that is enclosed by the dotted line, which is almost completely necrotic (Necr). Most nuclei formerly present in this region have been degraded, a process called karyolysis. Other areas where the nuclei from residual thymocytes have not been completely degraded continue to stain dark blue with hematoxylin. Arrow in B points to a Hassall body. C. Pan-cytokeratin (AE1/AE3) immunoreactivity. D. Cytokeratin 14 (CK14) immunoreactivity. Brown color indicates a positive reaction with antibody. Bar represents 1 mm in A and 500 μm in B–D.

**Fig 5:**
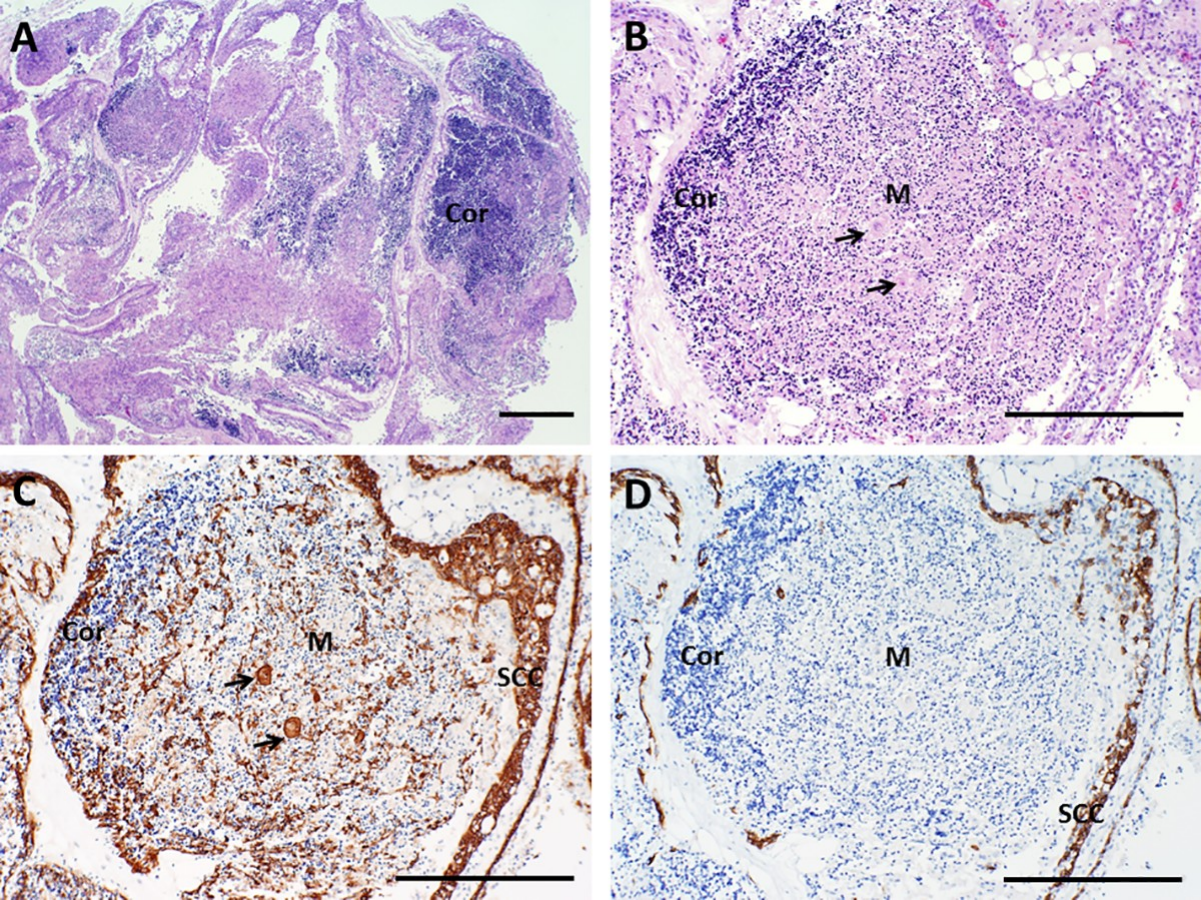
Architecture of cultured thymus, day 12. A, B. Hematoxylin and eosin (H&E) stain. At this time point, many thymocytes have either been lost from the tissue or they have died and their nuclei have been dissolved, making the tissue more eosinophilic (pink). Some areas retain architecture characteristic of normal uncultured thymus with cortical-like areas (Cor) that stain more basophilic (blue) and medullary-like areas (M), although with greatly decreased thymocyte cellularity. C. Pan-cytokeratin (AE1/AE3) immunoreactivity. D. Cytokeratin 14 (CK14) immunoreactivity. Brown color indicates a positive reaction with antibody. At this time point, the sub-capsular cortex (SCC) has thickened and epithelial cells appear more prominent due to the decreased numbers of thymocytes present. Arrows point to representative Hassall bodies. Bar represents 1 mm in A and 500 μm in B–D.

**Fig 6:**
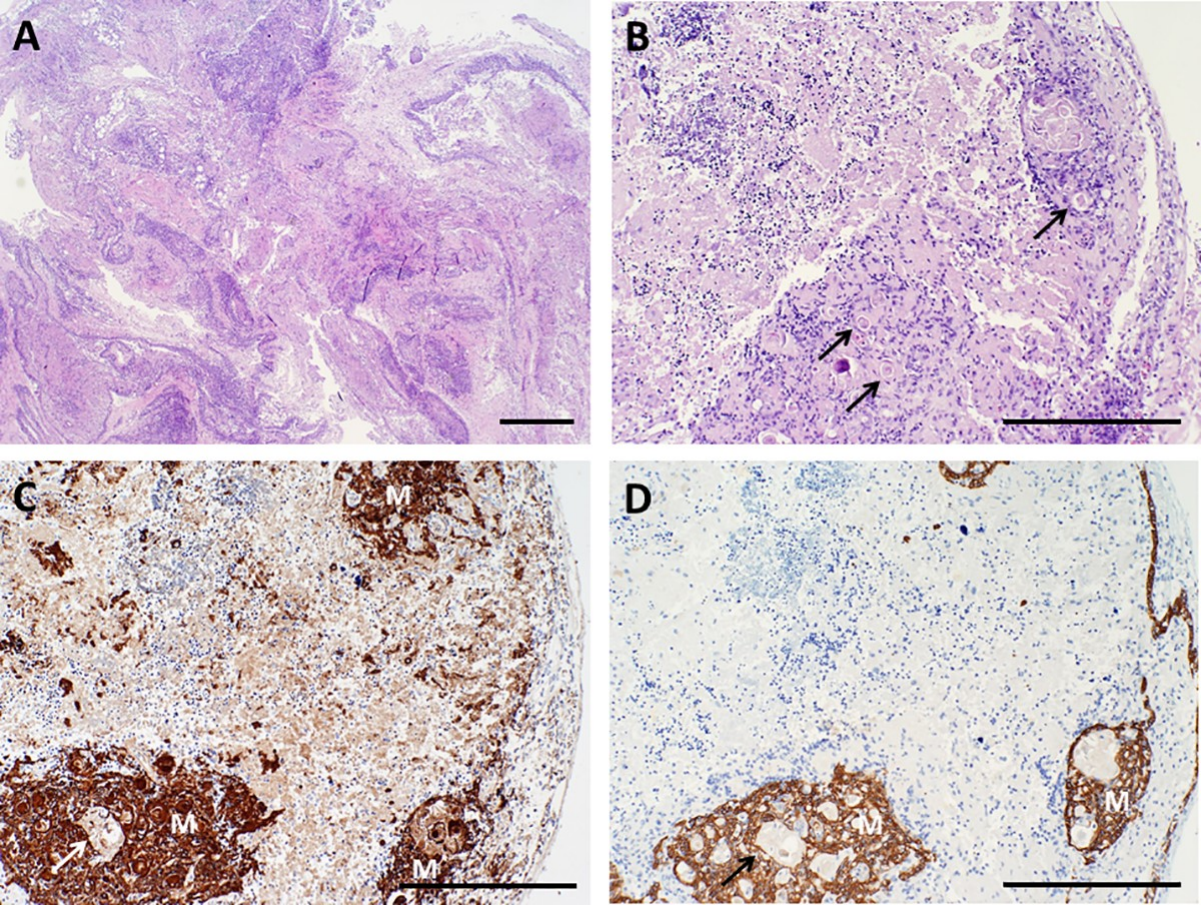
Architecture of cultured thymus, day 20. A, B. Hematoxylin and eosin (H&E) stain. At this time point, most thymocytes have either been lost from the tissue or they have died and their nuclei have been dissolved, making the tissue more eosinophilic (pink). Large groups of residual thymocytes are rare, although scattered cells with nuclear characteristics of thymocytes are evident. C. Pan-cytokeratin (AE1/AE3) immunoreactivity. Much of the epithelium present in formerly medullary areas (M) is condensed due to loss of medullary thymocytes, but scattered epithelial cells indicative of a residual light, lacy, three-dimensional network of thymic epithelial cells remain. D. Cytokeratin 14 (CK14) immunohistochemistry highlights former medullary areas and the subcapsular cortex. Brown color indicates a positive reaction with antibody. Arrows point to representative Hassall bodies. Bar represents 1 mm in A and 500 μm in B–D.

Thymocytes are progressively lost as thymus tissue is cultured, either from being flushed out during media changes or from thymocyte death followed by their degradation within the tissue (**Figs 3–6**). However, different from what occurs *in vivo*, dead cells may persist in cultured thymus long-term due to inability to recruit phagocytes to clear them. Thymocyte nuclei undergoing cell death may show pyknosis (chromatin condensation) and karyorrhexis (nuclear fragmentation) but typically, as these cells deplete their energy but are not phagocytosed, they show ragged nuclear edges with loss of nuclear membrane integrity. Karyolysis (complete dissolution of nuclei in necrotic cells) typically occurs within 2–3 days *in vivo*, but appears to occur more slowly during thymus culture. After 5–9 days in culture, most thymocytes have retained nuclei, but nuclear membranes are not intact, indicating early necrosis (**Fig 4**). These altered nuclear characteristics facilitate the distinction between viable and non-viable thymocytes, even when nuclei are retained. Large foci of eosinophilic necrotic cell debris where thymocyte nuclei have undergone karyolysis may also be seen (**Fig 4A**).

### Histologic assessment of cell integrity and tissue architecture

When cultured thymus tissue is to be transplanted, it is critical to be able to accurately distinguish between slices with extensive thymocyte necrosis but thriving thymic epithelial cells (the optimal tissue for transplantation) versus slices with similar amounts of necrosis where thymic epithelial cells are also compromised. Although histologic examination of standard H&E slides cannot directly assess viability, it can assess the degree of preservation of normal structures and the presence or absence of indicators of cell death. As described above, thymocytes are expected to be depleted during the culture process. Many thymocytes are washed from the slice during the daily media changes. Many others will die *in situ*, where their nuclei and cell bodies may remain for prolonged periods of time. However, the membrane integrity of dead thymocytes is compromised and their nuclei exhibit “ragged” edges. Thymocyte debris may clump together, making it impossible to discern individual cell borders. Thymocyte death and depletion is an expected and desirable consequence of culture and relative lack of intact thymocyte nuclei is potentially reflective of slice quality.

**Fig 7:**
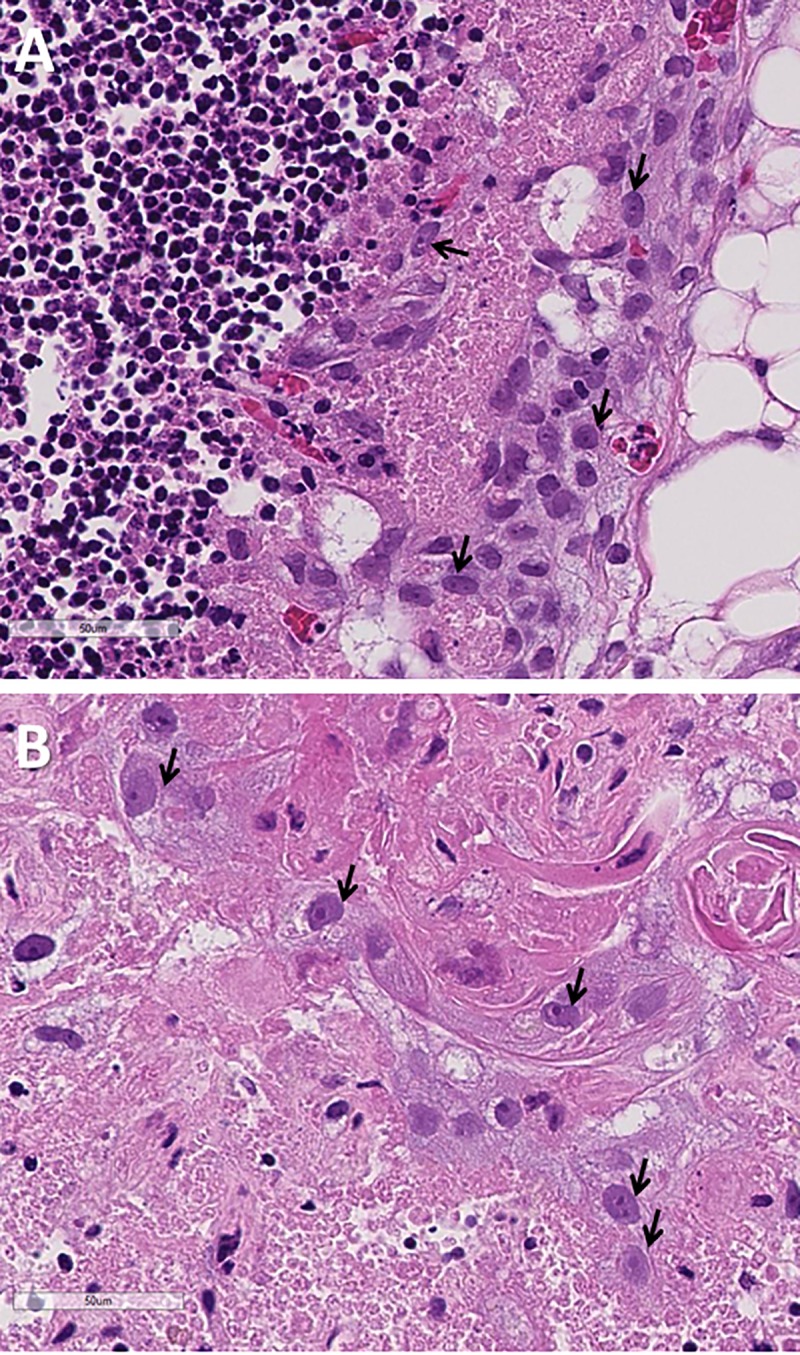
Assessing intact nuclei in thymus slices. Examples of intact thymic epithelial cell nuclei (arrows) are shown in the subcapsular cortex on day 9 (panel A) and in the medulla on day 21 (panel B). Hematoxylin and eosin stain; bar represents 50 μm.

The thymic epithelial cells are more easily visualized as thymocytes are depleted from the tissue. The nuclei of viable thymic epithelial cells are typically oval, larger than those of thymocytes, and have a sharply defined nuclear membrane outlined by the hematoxylin stain, with one or more nucleoli. These thymic epithelial nuclei typically look “open”, meaning they do not stain darkly with hematoxylin. This fits with an interpretation that they are alive and metabolically active, since active chromatin (“euchromatin”) does not bind the hematoxylin dye. The presence of nucleoli, which are the sites of ribosome synthesis, in many thymic epithelial cells further confirms that they were alive and metabolically active at the time of fixation. Examples of intact and viable-appearing thymic epithelial cells are shown in **Fig 7**.

Careful microscopic examination of multiple thymus slices at any given time point from day 0–21 showed that all slices derived from the same donor thymus closely resembled one another. The differences observed between different slices derived from the same thymus as a function of culture time were primarily related to the amount of necrosis (increased as culture time increased) and numbers of residual thymocytes (decreased as culture time increased). Slices derived from different donors were also qualitatively similar to each other when examined at similar time points. Differences between different donor tissues included relative size, shape, relative content of thymus versus medulla (but both were always present on each slice examined), amount of necrosis, condensation of thymic epithelium, and numbers of residual T cells. Most changes in histologic appearance had already occurred by day 5 of culture, with generally only additional depletion of thymocytes as cultures progressed.

### Assessment of thymic epithelial architecture

**Fig 8:**
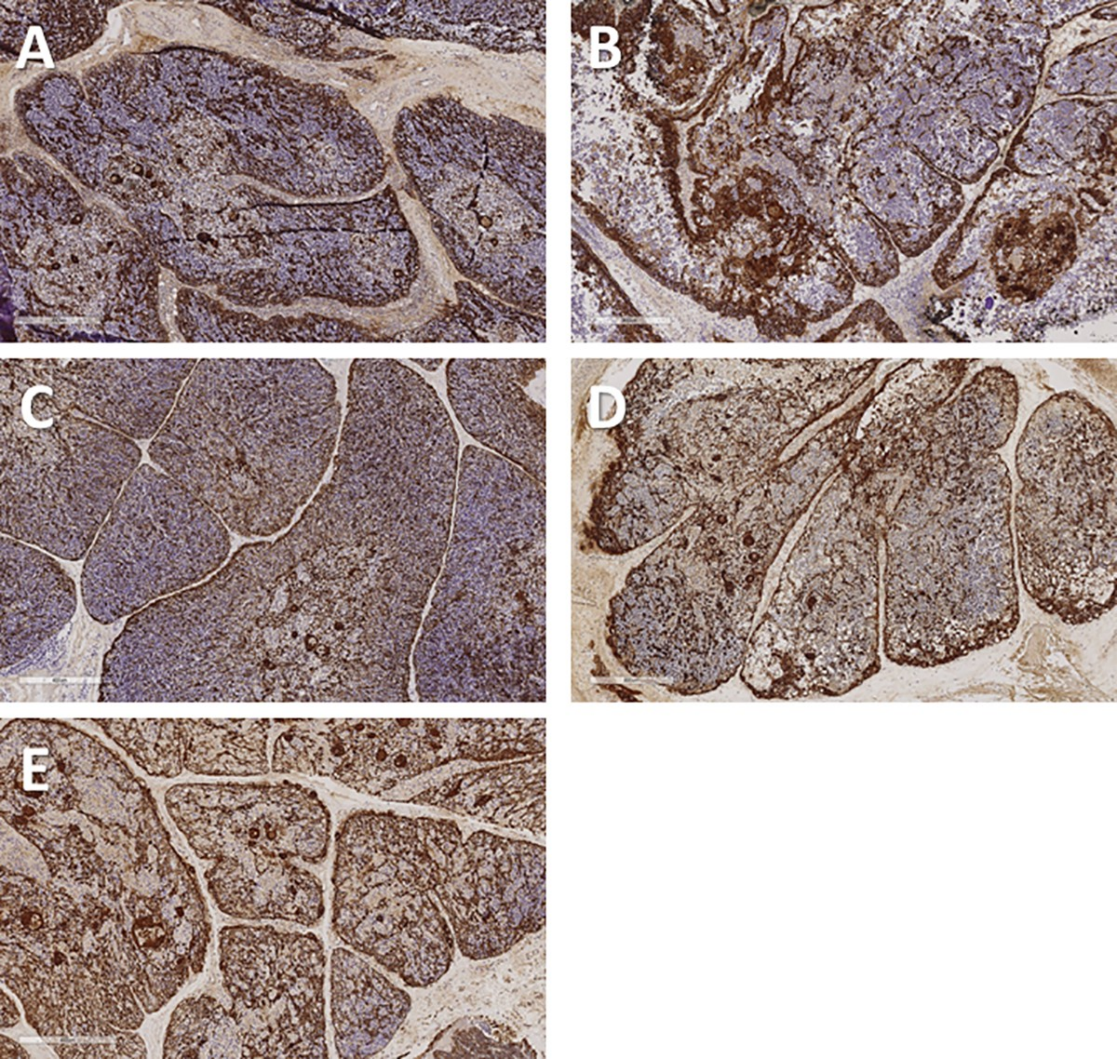
Comparison of thymic epithelial network of cultured thymus tissue at different time points. A. Day 0. B. Day 5. C. Day 9. D. Day 12. E. Day 21. Although there are time point-related differences in thymocyte depletion and the amount of necrosis such that the tissue become less basophilic (blue color) with time, the structure of the thymic epithelial network (brown color) remains intact as the culture progresses. Both cortical and medullary epithelium may condense as intervening thymocytes are depleted. Brown color indicates a positive reaction with a cocktail of anti-cytokeratin antibodies (AE1 + AE3); Hematoxylin counterstain. Bar represents 400 μm.

A cocktail containing anti-cytokeratin antibodies AE1 and AE3 detects essentially all thymic epithelial cells. In contrast, an antibody reactive with CK14 detects a subset of thymic epithelial cells that are present in the subcapsular cortex and seemingly scattered throughout the remainder of the cortex and medulla. Some of these CK14^+^ cells have been suggested to have the potential to differentiate into both cortical and medullary epithelial cells [17] and thus to represent long-term repopulating cells. As thymocytes are depleted from the tissue, the thymic epithelial cells become more visible. The three-dimensional thymic epithelial network is normally demonstrated in sections via a light and lacy arrangement of connected epithelial cells and/or seemingly scattered thymic epithelial cells whose connections are not evident in the section being examined. The three-dimensional thymic epithelial network contracts to varying degrees as thymocytes are lost during culture. This can result in condensation of the residual epithelium, such that the subcapsular cortical epithelial layer becomes thicker and medullary thymic epithelial cells become more tightly packed. Examples of these changes are shown in **Figs 1 and 3–6** for a single thymus lot examined on days 0, 7, 9, 12, and 20. Despite the loss of numerous thymocytes and potentially large areas containing necrotic debris, the overall architecture of the thymic epithelial cell network remains generally well-preserved through at least 21 days of culture (**Fig 8**).

### Assessment of residual T cells

**Fig 9:**
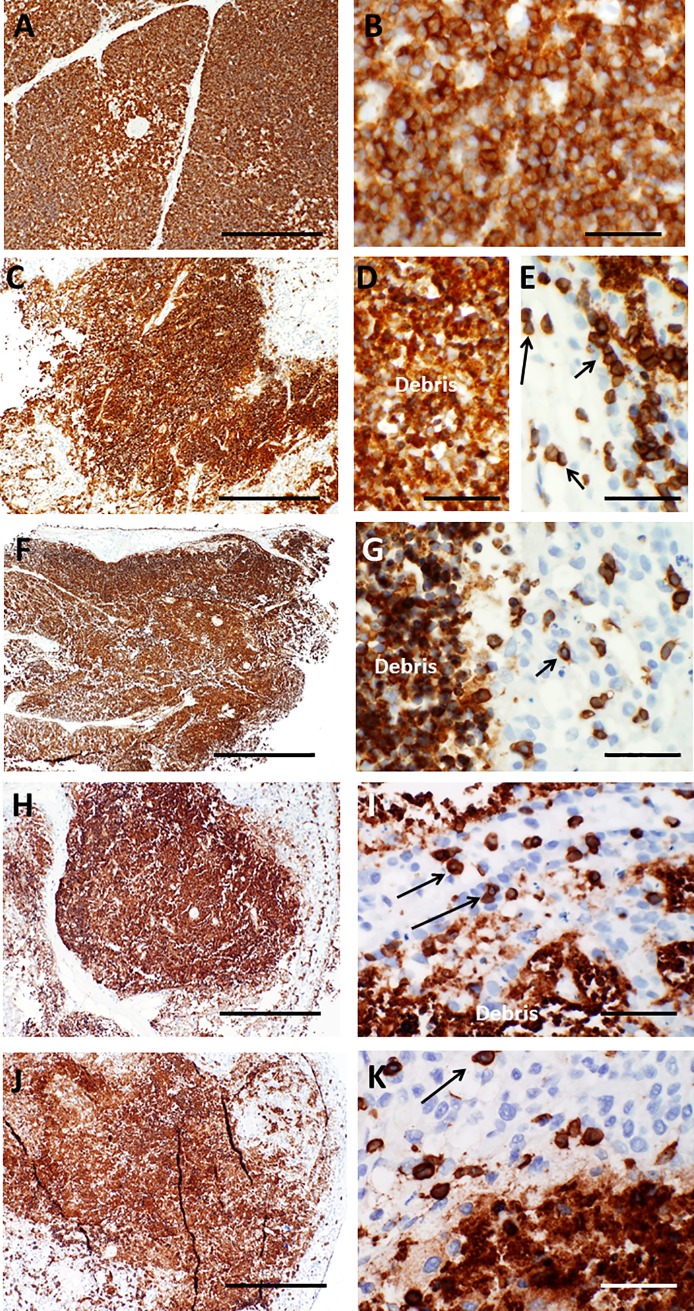
CD3 immunohistochemistry in thymus slices as a function of time in culture. A, B. On day 0, essentially all immature T cells in the cortex and more mature cells in the medulla react strongly with CD3 antibody. Higher magnification (B) shows pale blue nuclei surrounded by a ring of brown immunoreactivity, consistent with membrane expression of CD3. C—E. On day 7, the tissue still shows extensive reactivity with CD3 antibody. However, higher magnification (D, E) shows that the majority of the immunoreaction (brown color) is associated with debris from dead thymocytes, as most brown foci lack evidence of nuclei (D). Small foci of cells that demonstrate intact nuclei and membrane staining (arrows) can still be identified in areas away from the debris (E). As cultures progress through day 9 (F–G), day 12 (H–I), and day 21 (J–K), reactivity with thymocyte cellular debris remains strong, making it difficult to reliably detect potentially intact cells amidst the debris. The slices shown are all from a single lot that is representative of multiple lots examined at these time points. Bar represents 500 μm in panels A, C, F, H, and J and 50 μm in B, D, E, G, I, and K.

As described above, the primary purpose of culture of donor thymus tissues that are intended for transplantation is to partially deplete T cells to facilitate colonization of the transplanted slices with recipient thymocyte precursors and to decrease the risk of graft-versus-host disease. Immature T cells (thymocytes) and mature T cells can be identified in histologic sections by their morphology as well as via immunohistochemical stains that detect proteins specifically expressed by these cell types. CD3 is a component of the T cell receptor for antigen that is present on >95% of cortical and medullary thymocytes as well as all mature T cells. On day 0, the plasma membranes of essentially all immature T cells in the cortex and the medulla appear strongly reactive with CD3 antibody in a membrane pattern (**Fig 9A and 9B**). Viable thymocytes/T cells continue to react with CD3 antibody in a membrane pattern as cultures continue. However, the CD3 antibody also reacts strongly with dead thymocytes, including the anucleate debris that remains after dead thymocytes undergo karyolysis (**Fig 9D, 9G, 9I and 9K**). So much thymocyte debris remains that slices may still appear strongly and uniformly brown when viewed at low magnification (**Fig 9C, 9F, 9H and 9J**), although examination of the slide at higher power (e.g. 40X objective) confirms that relatively few intact potentially viable thymocytes are present at these time points. Non-viable cells and cellular debris will be cleared by recipient phagocytes after transplantation and therefore convey no risk of graft-versus-host disease. It is not generally possible to accurately identify CD3+ membrane immunoreactivity in tissue areas where considerable amounts of strongly staining debris prevents assessment of cellular membranes of adjacent cells. However, even at late time points during culture, CD3 immunohistochemistry typically highlights at least some thymocytes/T cells that have intact plasma membranes suggesting that they may still be viable (**Fig 9E, 9G, 9I and 9K**).

**Fig 10:**
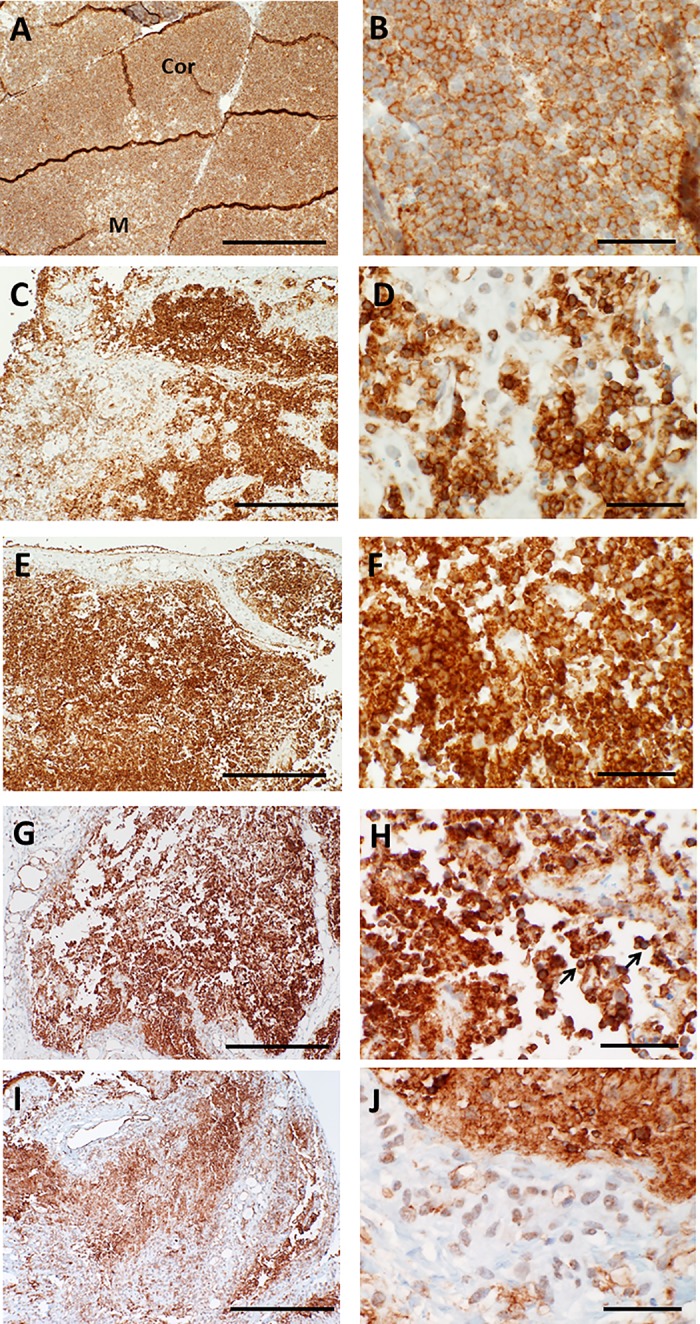
CD45RO immunoreactivity in thymus slices as a function of time in culture. A, B. On day 0, essentially all immature T cells in the cortex (Cor) and medulla (M) react strongly with antibody specific for CD45RO. Higher magnification (B) shows the strong brown positive membrane reactivity. C, D. By day 7, reactivity with CD45RO antibody is strongest in the medulla. Some positive cells exhibit the expected specific membrane staining, but there is also considerable reactivity with cellular debris. Loss of membrane immunostaining and intense reactivity primarily with residual thymocyte debris is seen on days 9 (E, F), 12 (G, H) and 21 (I, J), although some cells with intact membrane staining can occasionally still be detected (arrows in H). Bar represents 500 μm in panels A, C, E, G, and I and 50 μm in B, D, F, H, and J.

**Fig 11:**
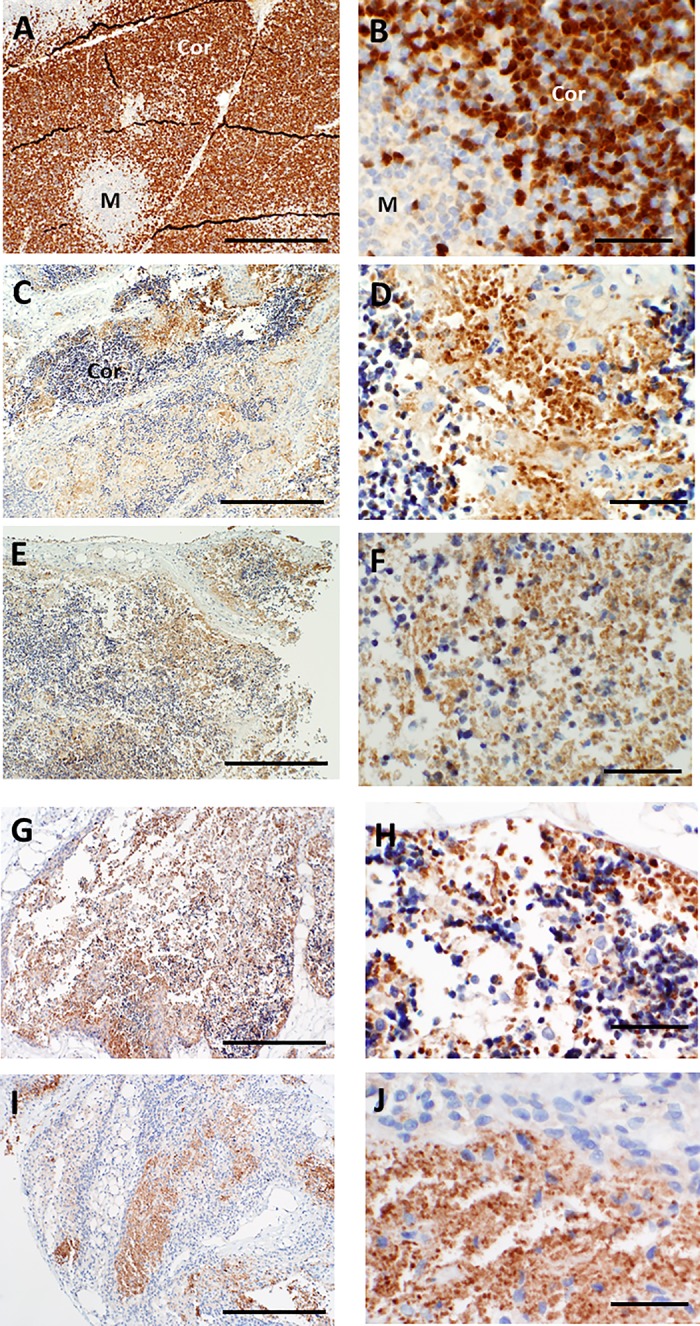
TdT immunoreactivity in thymus slices as a function of time in culture. A, B. On day 0, essentially all immature T cells in the cortex (Cor) react strongly with antibody specific for TdT. Higher magnification (B) shows strong brown positive reactivity with the nuclei of cortical thymocytes with rare positive cells in the medulla (M). C, D. By day 7, the nuclei of most thymocytes that remain in cortical areas are small and strongly basophilic (blue) consistent with apoptosis and they fail to react with TdT antibody. The antibody reactivity shown in the center of D is with cellular debris. Similar lack of TdT immunostaining of residual thymocyte nuclei is seen on days 9 (E, F), 12 (G, H) and 21 (I, J). Bar represents 500 μm in panels A, C, E, G, and I and 50 μm in B, D, F, H, and J.

**Fig 12:**
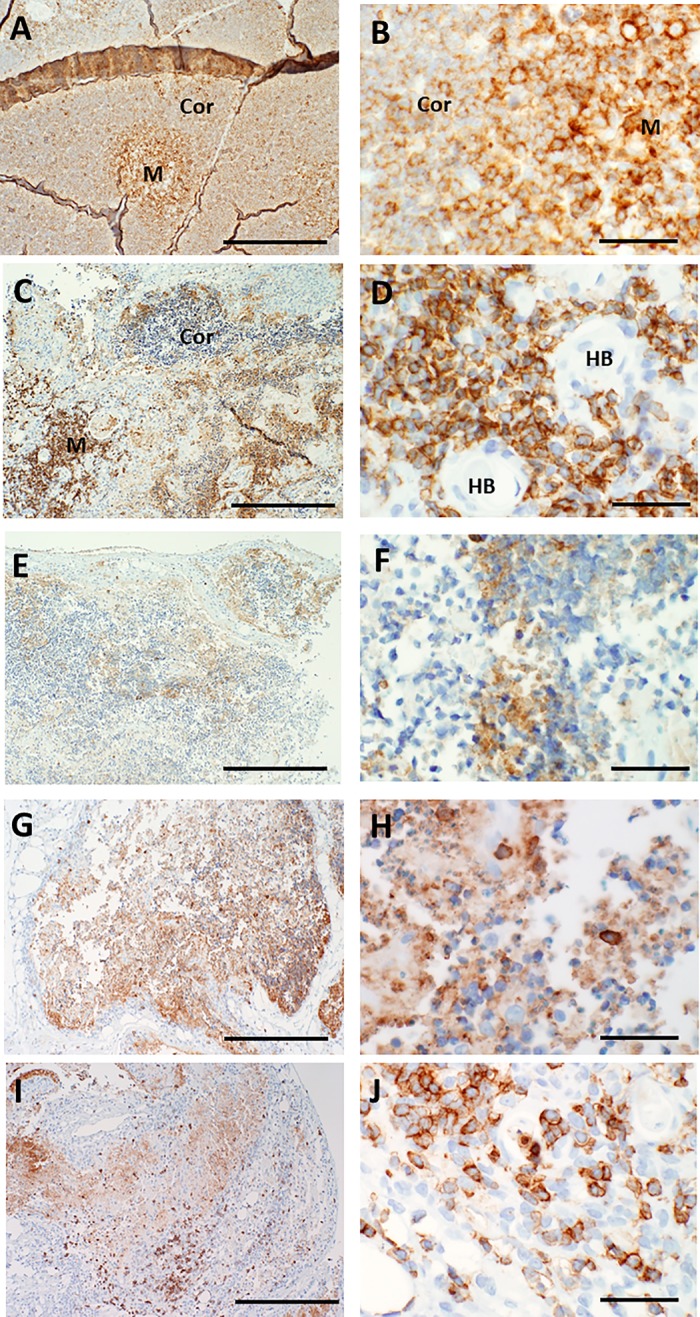
CD43 immunoreactivity in thymus slices as a function of time in culture. A, B. On day 0, immature thymocytes in the cortex (Cor) react weakly and those in the medulla (M) react more strongly with antibody specific for CD43. The dark bands in A are sectioning artifacts (wrinkles and folds in the tissue). Higher magnification (B) shows the brown positive membrane reactivity with both cortical and medullary thymocytes. C, D. By day 7, reactivity with CD43 antibody is generally limited to the medullary thymocytes. Some positive cells exhibit the expected specific membrane staining, but there is also considerable reactivity with cellular debris in cortical areas. Loss of membrane immunostaining and intense reactivity primarily with residual thymocyte debris is seen on days 9 (E, F), 12 (G, H) and 21 (I, J), although some cells with intact membrane staining can be detected at these later time points. Bar represents 500 μm in panels A, C, E, G, and I and 50 μm in B, D, F, H, and J.

**Fig 13:**
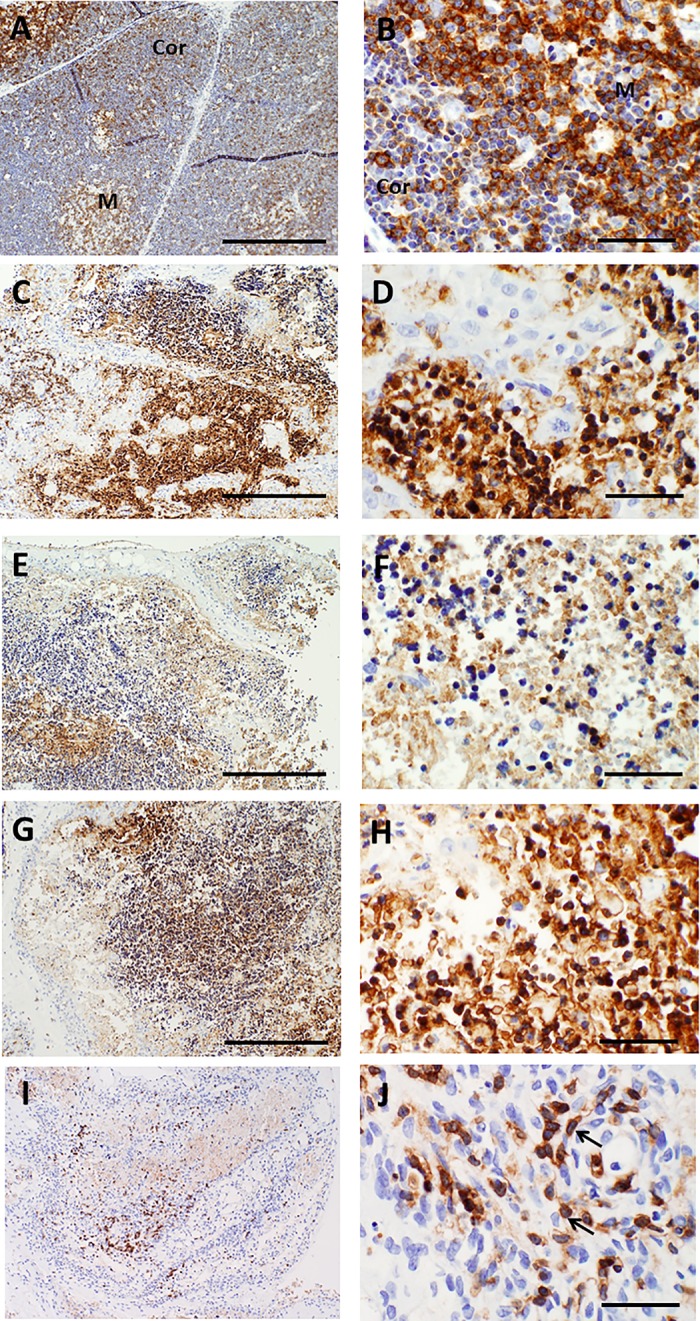
βF1 immunostaining in thymus slices as a function of time in culture. A, B. On day 0, a subset of immature T cells in the cortex (Cor) and essentially all of the medullary thymocytes react strongly with βF1 antibody that is specific for the β chain of the αβ T cell receptor. Higher magnification (B) shows strong brown positive membrane reactivity; M denotes medulla. C, D. Reactivity with antibody remains strong on day 7, however the specific membrane staining is lost and reactivity is primarily with cellular debris in medullary areas. Similar loss of membrane immunostaining and reactivity primarily with residual thymocyte debris is seen on days 9 (E, F), 12 (G, H) and 21 (I, J), but cells with intact membranes can be detected (see arrows in J). Bar represents 500 um in panels A, C, E, G, and I and 50 um in B, D, F, H, and J.

To determine whether other thymocyte-reactive antibodies that were currently validated by our laboratory for clinical diagnostic use could better identify residual immature or mature T cells in cultured thymus tissues, patterns of immunohistochemical reactivity were qualitatively assessed for cultured thymus reacted with antibodies that recognized CD45RO, terminal deoxynucleotidyl transferase (TdT), CD43, and the beta chain of the T cell receptor (βF1). As shown in **Figs 10–13**, CD45RO antibody had a similar pattern of reactivity with cortical versus medullary thymocytes as CD3 antibody, while fewer overall thymocytes were reactive with TdT, CD43, and βF1 antibodies. However, TdT, CD45RO, CD43, and βF1 antibodies also reacted with thymocyte debris, and use of these antibodies did not improve identification of the small numbers of intact-appearing residual thymocytes late in culture.

### Assessment of cellular proliferation

**Fig 14:**
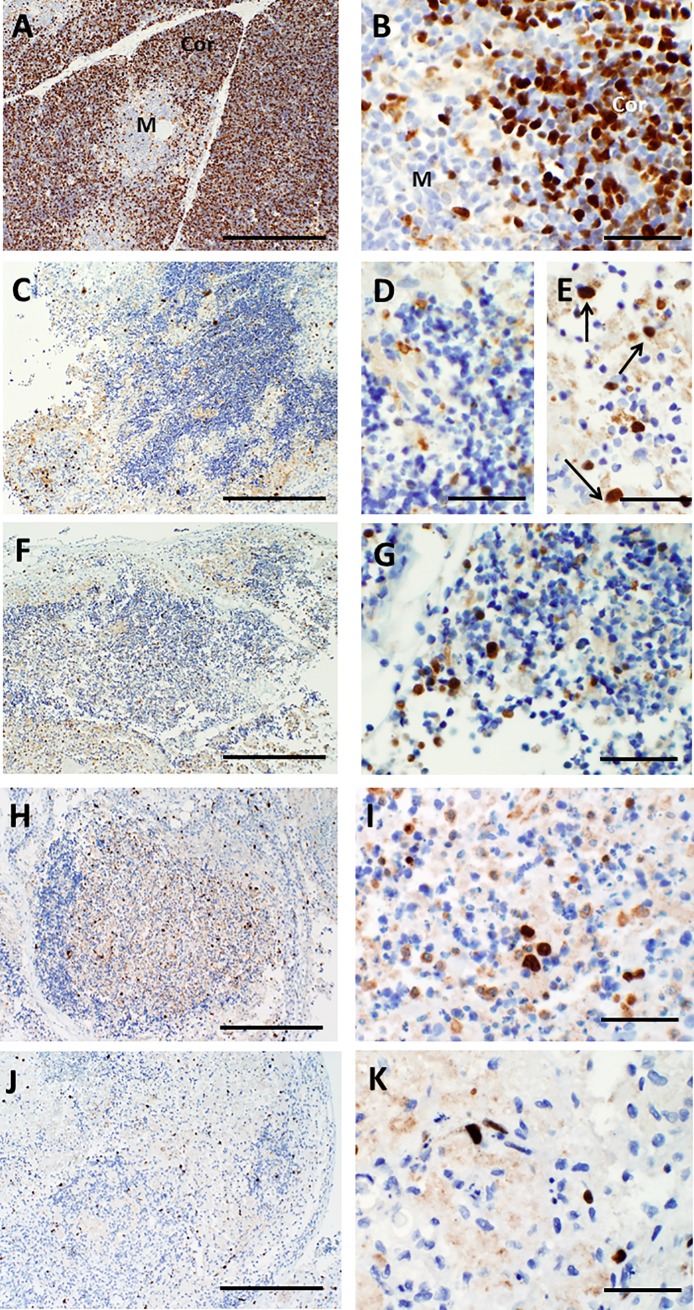
Ki-67 immunohistochemistry in thymus slices as a function of time in culture. A, B. On day 0, the nuclei of the majority of immature T cells in the cortex (Cor) react strongly with antibody specific for Ki-67. Higher magnification (B) shows strong brown positive reactivity with the nuclei of cortical thymocytes. Only rare lymphocytes in the medulla (M) react with antibody. C- E. By day 7, the nuclei of most thymocytes that remain in cortical areas are small with indistinct nuclear borders consistent with apoptosis, and they fail to react with Ki-67-specific antibody (D). The cells that react with antibody (E, arrows) have larger nuclei that suggest they are thymic epithelial cells. Similar lack of Ki-67 labeling of residual thymocyte nuclei is seen on days 9 (F, G), 12 (H, I) and 21 (J, K). The slices shown are all from a single lot that is representative of multiple lots examined at similar time points. Bar represents 500 μm in panels A, C, E, G, and I and 50 μm in B, D, F, H, and J.

The Ki-67 antigen is expressed in the nucleus of all cells that are proliferating (i.e. not in the G0 phase of the cell cycle). On day 0, the majority of the immature thymocytes present within the thymic cortex are proliferating and their nuclei react strongly with antibody specific for the Ki-67 proliferation antigen. In contrast, only rare more mature medullary thymocytes and/or epithelial or stromal cells react with this antibody (**Fig 14A and 14B**). The pattern of immunoreactivity observed after day 1–2 of culture is of scattered rare positive cells, almost all of which have the larger nuclei characteristic of thymic epithelial cells (**Fig 14C–14K**). Thus, Ki-67 reactivity may be a useful adjunct to nuclear appearance by H&E for documenting the viability of thymic epithelial cells within the slice.

### Validation of histopathologic assessment criteria using clinical thymus lots

From 1995 through 2019, qualification of cultured thymus lots for transplantation has simply required documentation of sterility (lack of bacterial and mycoplasma contamination) and fulfillment of simple qualitative histopathologic diagnostic criteria that identify the tissue as thymus and document its viability and tissue organization (**Tables 1 and 2**). Careful microscopic examination of the 8 research lots submitted *in toto* for histologic examination showed that different slices derived from the same or different thymus tissue were qualitatively similar and were ranked identically per **Tables 1 or 2** at any given time point in culture. Differences between slices derived from the same donor thymus were limited to relative size, shape, content of cortex versus medulla (but both were always present on each slice examined), amount of necrosis, condensation of thymic epithelium, and numbers of residual T cells. Furthermore, since the major change that occurred between days 5–9 and 12–21 was progressive depletion of thymocytes, the tissue histology on day 5–9 was reflective of that observed on days 12–21, just with more thymocyte depletion at the later time points.

**Table 1:** Diagnostic criteria for thymus submitted on day 0*.

Assay	Acceptance Criteria	Result Required for Acceptance
Identity	50% of sampled tissue contains keratin AE1/AE3 networks in lacy staining pattern	Present
Potency	Hassall bodies identified; CK14 staining in lacy pattern	Present
Viability	>90% intact nuclei observed	Present

*Any additional immunostains performed (e.g. CD3 or Ki-67) should also be commented upon.

**Table 2:** Diagnostic criteria for thymus that has been cultured for one or more days*.

Assay	Acceptance Criteria	Result Required for Acceptance
Identity	Areas positive for keratin AE1/AE3	Present
Potency	At least one Hassall body identified; CK14 staining scattered throughout	Present
Viability	Intact thymic epithelial nuclei observed	Present

*Any additional immunostains performed (e.g. CD3 or Ki-67) should also be commented upon.

Retrospective review was performed for 11 cultured thymus lots that were processed either contemporaneously with or up to 2.5 years prior to these research lots then transplanted into athymic recipients. All 11 lots met the diagnostic criteria shown in **Tables 1 and 2** for slices examined on culture days 0, 5–10, and 12–21. The histologic appearance of each of these slices (n = 33) closely resembled that of the research lots examined at a similar time point, with no substantive deviations from the detailed descriptions above. Of note, all 11 recipients developed sufficient T cell reconstitution to resist infections, with naïve CD4 T numbers ranging from 74 per mm^3^ to normal for age, when tested 1–3 years post-implantation of cultured thymus.

## Discussion

This study describes histopathologic changes that occur when postnatal human thymus is cultured for up to 21 days. As thymic cultures progressed, slices developed increasing amounts of necrosis, increasing condensation of thymic epithelium, and decreasing numbers of residual T cells. The thymic epithelial network remained intact throughout the 21 days of culture, with continued expression of cytokeratin 14, a putative biomarker of thymic epithelial cells with long-term organ-repopulating potential. Slices from the same thymus were qualitatively similar, such that a single slice could adequately represent the entire thymus. Variability in histologic appearance of cultured thymus slices derived from different donors was also minimal at any given time point. The tissue histology observed early during culture (e.g. days 5–9) closely reflected what was observed later in culture (e.g. days 12–21), although more thymocyte depletion and necrosis were observed at the later time points.

Immunohistochemistry for antibodies that recognize CK14 or all types of cytokeratins (AE1/AE3) was helpful in evaluation of cultured thymus slices. Cytokeratin intermediate filaments are important components of the cytoskeleton of all epithelial cells. The pan-cytokeratin AE1/AE3 stain demonstrates the epithelial network within the examined thymus slice, which may not be easily discernable at later time points using H&E stain alone. The specific type of cytokeratin expressed by a particular thymic epithelial cell has been shown to depend on its stage of development and differentiation and functional state. In mice, antibodies that react with CK5, CK8, and CK14 have been most commonly used to identify thymic epithelial cell subtypes [17]. Expression of CK14 was determined in this study, based on previous studies where CK14 expression was hypothesized to be a characteristic of thymic epithelial cells with the potential to differentiate toward either cortical or medullary lineages [18]. While the clinical outcome of long-lasting immune reconstitution [5] suggests that thymic epithelial progenitors are likely present within transplanted thymus slices, the precise phenotype for such progenitors has not been definitively identified in humans. The phenotype of thymic epithelial progenitors in mice is still controversial [19–22] and only low numbers have been detected using multiple antibodies and multi-color flow cytometry. Thus direct detection of thymic epithelial progenitors in human tissue sections is not currently feasible.

These studies identified potentially large amounts of necrotic cellular material remaining within thymic slices at culture days 12–21, the time points when the tissue might be transplanted. Biopsy examination several months after transplantation shows no evidence of this necrotic material [11]. We hypothesize that the initial presence of this necrotic debris and its underlying extracellular matrix at the time of transplant may serve to preserve the functional architecture of the slices, including the cortical and medullary niches for developing thymocytes. Experimental use of thymus decellularization approaches that preserve extracellular matrix to recreate murine thymic “organoids” that can support cellular differentiation of both epithelial cells and hematopoietic precursors [23] supports this hypothesis.

Previous studies demonstrated that the growth potential of thymic epithelial cells was robustly maintained under the culture conditions used, such that cyokeratin-positive epithelial monolayers could be established from the slices up to 12 weeks after initiation of organ culture [10]. The current study did not directly assess epithelial cell viability, but instead used the presence of intact nuclei as a surrogate marker. Since the histopathologic examination focused on evaluation of cellular and tissue structures that could be readily discerned by staining with H&E or reactivity with cytokeratin antibodies, it was inherently limited by the inability of these stains to directly measure viability and cellular function. Thus, histopathologic examination using only H&E stains combined with immunohistochemistry for cytokeratins cannot necessarily address all factors that may influence the quality of the cultured thymus slices. A positive reaction of thymic epithelial cell nuclei with antibodies that recognize the Ki-67 proliferation antigen provides proof that those cells are alive, since Ki-67 is only expressed in actively proliferating cells, i.e. not in G0 phase. However, the sensitivity of this approach is likely limited by the relatively small percentage of thymic epithelial cells that are proliferating at any given point in time.

We also note that, based on our studies to date, it is not possible to link overall patient survival or the time required for their immunoreconstitution with specific histopathologic characteristics of the specific lot of cultured thymus received. Viable-appearing thymus allograft has been identified post-mortem in at least one patient who died post-transplantation [11]. Patient factors such as severe congenital malformations or infections can lead to death prior to T cell reconstitution. Furthermore, clinical requirements for allograft-damaging therapy such as prolonged high-dose corticosteroids may prevent function, independent of thymus lot quality. Studies to identify additional biomarkers that track with thymic epithelial viability and potential function are on-going and will help to definitively address the issue of thymus allograft quality at implantation versus adverse clinical condition in governing the survival and immune reconstitution of transplanted patients.

In summary, histopathologic examination using H&E and cytokeratin immunoreactivity can be useful for assessment of cultured thymus slices. The value of the histopathologic examination to prospectively assess the quality of slices intended for transplantation into patients with congenital athymia may potentially be improved by identifying and developing assays for new biomarkers that reflect thymic epithelial cell viability and/or function.
